# Effects of Deacidification on Composition of *Schisandra chinensis* Ethanolic Extract and Studies on Acute Toxicity in Mice

**DOI:** 10.3390/molecules25246038

**Published:** 2020-12-21

**Authors:** Gaosheng Hu, Zhuangbo Qi, Anhua Wang, Jingming Jia

**Affiliations:** 1 School of Traditional Chinese Materia Medica, Shenyang Pharmaceutical University, Shenyang 110016, China; 103040307@syphu.edu.cn (G.H.); qizhuangbo@163.com (Z.Q.); 2 China-Korea Joint Laboratory of Molecular Pharmacognosy, Shenyang Pharmaceutical University, Shenyang 110016, China

**Keywords:** *Schisandra chinensis*, deacidification, organic acid, lignan content determination, acute toxicity

## Abstract

*Schisandra chinensis Fructus* (SCF), a well-known traditional medicinal material, is a rich source of dibenzocyclooctene type lignans and polyphenols, which are important ingredients in SCF and show various activities. SCF also contains about 18% organic acids, mainly citric acid, which makes the fruit and extract taste extremely sour and limited its application in beverages or food industries. In the present study, a chemical deacidification method was applied to defatted and non-defatted ethanol extract of SCF, and the effects on organic acid, lignans, and phenolic compounds were evaluated. Free radical scavenging activity and acute toxicity in mice before and after deacidification were also compared. Our results demonstrated that chemical deacidification significantly decreased the contents of organic acid and lignan compounds and markedly improves the safety of the ethanol extract of SCF, which will facilitate the comprehensive utilization of SCF extract in food and beverage industries.

## 1. Introduction

*Schisandra chinensis* (Turcz.) Baill, belonging to the family of Magnoliaceae, is distributed mainly in Northeast of China, Russia, Korea Peninsula, and Japan [1]. Its dried ripe fruit, *Schisandra chinensis Fructus* (SCF), is named Wuweizi in Chinese, meaning fruits with five flavors: Sour, bitter, sweet, spicy, and salty. The ripe fruits are usually harvested late September in China. It has been used mainly for the treatment of night sweat, insomnia, and thirst due to insufficiency of body fluid and frequent urination since the ancient times of Shen Nong’s Herbal Classics [2]. In the official list given in the China Pharmacopeia (2015 edition), there are 92 prescriptions using *S. chinensis* as an ingredient [2], which means that this red fruit has been used widely in the Chinese pharmaceutical industry. A few foods and beverages are using this fruit as their main ingredient, but their market percentage share remains very low and they are hardly found in market.

It was reported that the fresh ripe fruit contains 3.26% ± 0.06% citric acid, 1.13% ± 0.04% malic acid, and 0.53% ± 0.01% shikimic acid [3]. Considering the drying rate of about 20% [4], the contents of three acids in dry fruit should be about five times of their contents in fresh fruits, which will make the fruit taste extremely tart, much stronger than lemon (citric acid content is approximately 8% in dry lemon) [5]. The high citric acid content and the accompanying tartness limited its consumption by the food and beverage industry and related markets. As reported, the high concentration of organic acids and low pH is associated with the dissolution of dental enamel when consuming fruit type beverages [6]. Besides, acidic foods are also believed to trigger the gastroesophageal reflux disease (GERD) symptoms or make symptoms worse [7,8]. Considering its high organic acid content, it is important to decrease the content of organic acid in order that flavored foods or beverages can be developed from *S. chinensis*. Deacidification is a useful procedure to improve fruit juice quality, especially when there is a need to increase the sugar-acid ratio [9]. There are mainly four kinds of deacidification method: (1) The chemical method, where applying calcium carbonate to the concentrated juice and the organic acid is precipitated and removed by filtration; (2) the physical method, where organic acids were removed by passing the extract or juice through anion exchange resin; (3) the biology method, by fermentation using lactate favoring microorganisms, which is often used in the grape wine preparation process; and (4) electrodialysis, which introduces and increases the sodium concentration [9]. It was reported that the polyphenol compounds retention rate was 35.32–70.53% using different anion exchange resins (final pH = 4.0) and 91.77 when calcium carbonate addition method was used in fresh SCF pulp juice (final pH = 4.68) [10]. According to these results, the calcium carbonate method was adopted in this study.

It has been proven that dibenzocyclooctene type lignans are one of the most important active compounds [11,12] and show activities like hepatoprotective [13], sedation [14], protection against Alzheimer’s disease [15], anti-cancer, anti-diabetic, anti-aging, and anti-obesity activities, as reviewed in a comprehensive publication [16]. Among the lignans, the content of schisandrin has been used for the quality control in China Pharmacopeia [2]. These lignans are usually low polarity compounds and can be enriched by non-polar solvents like *n*-hexane [17]. The seeds of SCF are a rich source of fatty acid (about 14.1% DW) [18], which can also be extracted by *n*-hexane.

Besides, this fruit also contains polyphenol compounds, mainly proanthocyanidins in the seed coat [19] and anthocyanin in the pulp tissue [20,21] with antioxidant activity. In our previous optimization experiment, we found the maximum polyphenolic compounds can be extracted when 60% ethanol was used as solvent [18]. In the present study, 60% ethanol was used to prepare different SCF samples. Therefore, to establish a method to decrease the content of total organic acid in ethanolic extract of SCF, the effects of this process on the contents of other compounds must be evaluated.

In the present study, a chemical deacidification method was used to eliminate organic acids from the ethanol extract of SCF. The levels of total organic acid, lignan compounds, phenolic compounds, and free radical scavenging activity were determined using potentiometric titration, HPLC analysis, spectrophotometry, and DPPH assay, respectively. Besides, the acute toxicity of deacidified and non-deacidified extracts was compared in mice.

## 2. Results and Discussion

### 2.1. Content Determination of Organic Acid in A, B, C, and D

As indicated in Table 1, in defatted extracts, 86.29% organic acid was eliminated from sample A to obtain sample B. Similarly, 88.00% organic acid was eliminated from sample C, and sample D was obtained. Our results demonstrated that the deacidification method can decrease the organic acid remarkably in both defatted and non-defatted ethanol extract of SCF.

This method was previously used in citric acid purification, in which the calcium citrate is precipitated and recovered by filtration, and sulfuric acid is then added to convert this precipitate into citric acid and calcium sulfate. By subsequent filtration, the citric acid can be recovered and purified [22]. In the deacidification process used in the present study, after addition of an excess amount of calcium carbonate, the citric acid reacts to give calcium citrate, which is a precipitate in water or ethanol solution. The precipitate can be removed by vacuum filtration. However, due to the excess amount of calcium carbonate, calcium bicarbonate will also be produced, which is insoluble in ethanol, but soluble in water (16.6 g/100 mL, 20 °C). Therefore, subsequently, 1.5 volume of 95% ethanol was added to this solution, and calcium bicarbonate was precipitated and removed by vacuum filtration using a Buchner funnel coupled with a vacuum pump.

As reported, the main organic acid in SCF is composed mainly of citric acid, which is soluble in water and ethanol. In the preparation of SCF extract, the citric acid will be extracted with other main active compounds, such as lignans and phenolic compounds. Therefore, the effects of this deacidification process on the lignan and phenolic compounds were investigated.

### 2.2. Effects of Deacidification on the Contents of Lignan of Different SCF Extract

**Figure 1:**
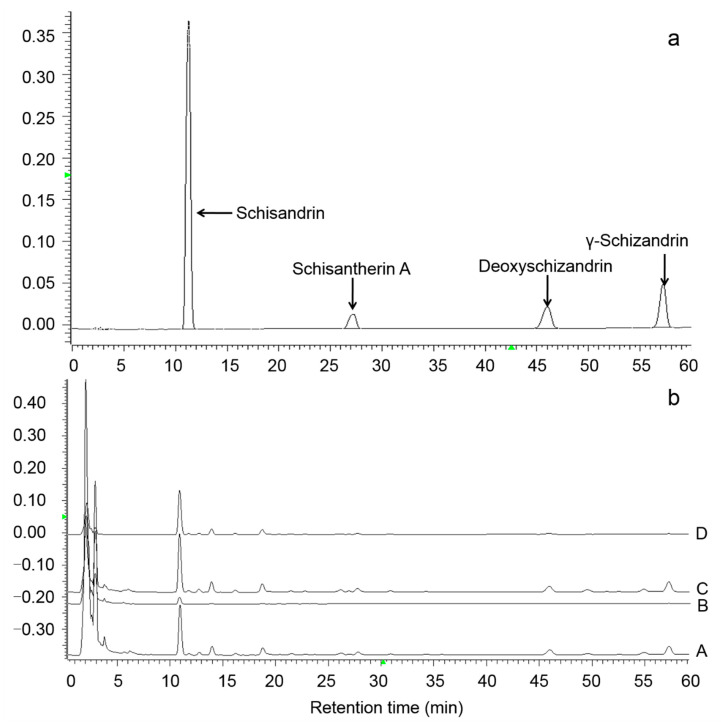
HPLC chromatograms of four lignan standard compounds (**a**) and the four *S. chinensis* extract samples A, B, C (diluted five times), and D (diluted five times) (**b**).

As shown in Figure 1, the four lignan standard compounds are well separated under current HPLC condition and can be used for quantification of the ethanol extracts. Due to the hexane extraction in samples A and B, the lignan contents of samples C and D were several times higher than that of samples A and B.

**Figure 2:**
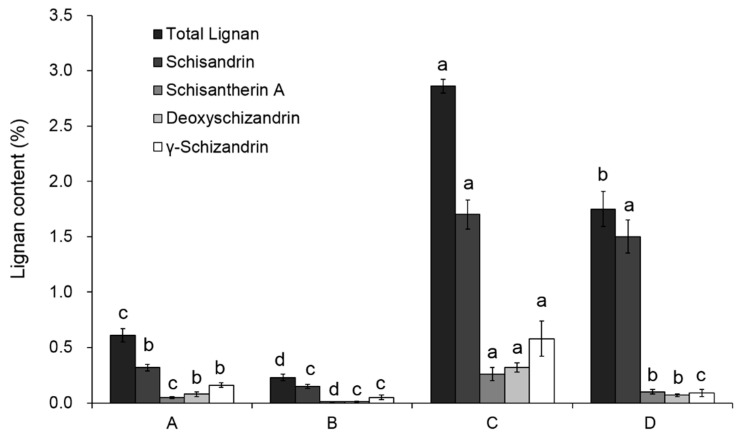
Determination of the total and individual lignan contents in samples A, B, C, and D. Note: The different letters above columns represent the significance of each index (*p* < 0.05) among different samples.

As shown in Figure 2, schisandrin was the most abundant lignan compound in four samples, accounting for 59–85% of the total lignan. After the deacidification process, the ratio of schisandrin to total lignans increased, while the ratio of the other three lignans decreased. This might be due to the poor polarity and solubility of Schisantherin A, deoxyschizandrin and γ-schizandrin in the deacidification process. By comparing the lignan content of samples A and C, it is evident that nearly 80% of the total lignan is removed by the hexane extraction procedure. After the deacidification process, the lignan loss was also observed. As shown in Table 2, about 70.6 percent of the total lignan from sample A was lost when preparing sample B, and 38.32 percent was lost in the deacidification procedure from C to D. In sample C, the lignan can be extracted with polyphenolic compounds and the total content of lignan in SCF reached 1.14%, which is similar with published results [18].

### 2.3. Determination of Total Phenolic Compounds, and DPPH Scavenging Activity

**Figure 3:**
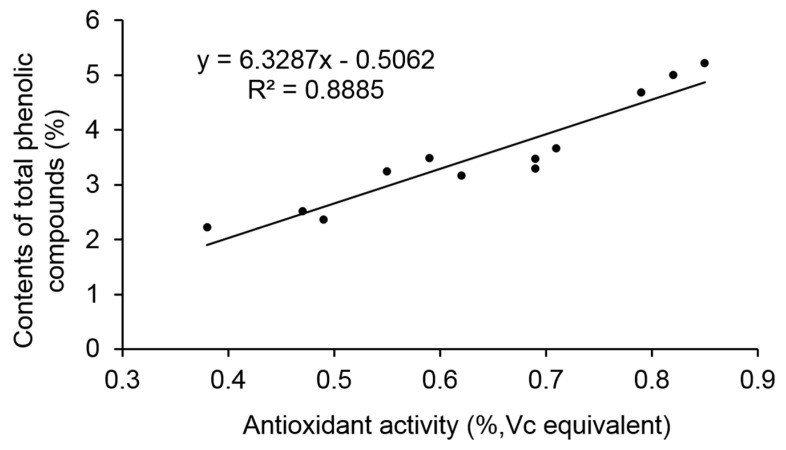
Linear correlation analysis between antioxidant activity and contents of total phenolic compounds in different samples.

As indicated in Table 2, total phenolic and free radical scavenging activity (presented by Vc equivalence) in the deacidification process were also affected markedly. It was also reported that, the main phenolic included proanthocyanidins and anthocyanin in SCF ethanol extract. The loss might be due to the low solubility of their reaction product with calcium carbonate. In sample C, the content of total phenolic compounds in SCF was 119.61 mg/g SCF, which is similar with our previous determination results of 104.24 mg/g DW SCF [23]. Polyphenol compounds are important nutrient elements and the total phenolic compounds are main substance basis of antioxidant activity in SCF [23]. Therefore, the effects of deacidification on the phenolic compounds provide essential information for the evaluation of the process. It is obvious that the total phenolic compounds loss rate (27.21 ± 0.04%) in B compared with A was about half of that in D compared with C (55.92 ± 0.05%), which also demonstrated that the preparation process of sample B is more feasible. Besides, in accordance with other publications, a strong correlation (R^2^ = 0.8885) between antioxidant activity and total phenolic compounds is observed (Figure 3).

### 2.4. Maximal Tolerance Dose and Maximal Feasible Dose of A, B, C and D in Mice

It was reported that the ethanol extract of *S. chinensis* was toxic to mice by oral administration, and the calculated LD_50_ of the SCF is 14.67–19.96 g/kg body weight (BW) [24]. Here we summarized the reported LD_50_, the content in *S. chinensis*, and the calculated LD_50_ of SCF of two main lignans (schisandrin and γ-schizandrin), essential oils, citric acid, and the ethanol extract when orally administered in mice. As shown in Table 3, the lignan compounds (schisandrin and γ-schizandrin) showed the lowest LD_50_ of 1.0 and 0.25 g/kg BW. However, considering their content in SCF, the corresponding LD_50_ calculated was several times higher than the ethanol extract. The SCF corresponding LD_50_ of essential oils was also dozens of times higher than that of ethanol extract. However, the SCF equivalent LD_50_ of citric acid (28 g/kg BW) is closest to that of the ethanol extract (14.67–19.96 g/kg BW) of *S. chinensis*, which indicated the citric acid might be related with the found toxicity in mice.

As mentioned in Table 1 and Table 2, the contents of organic acid and total lignan decreased significantly after the deacidification procedure. Therefore, the acute toxicity experiment was carried out in mice by oral administration.

Our results (Table 4) showed that there were no deaths or significant behavioral changes found in the animals that received B at a volume of 0.2 and 0.3 mL/10 g BW. Furthermore, no deaths or any significant behavioral changes were observed in the animals that received D at a volume of 0.2 mL/10 g BW; however, 80% of mice died when the volume of D was increased to 0.3 mL/10 g BW. Considering the analytical results in Table 2, higher lignan content might be the crucial factor.

The mortality rate of mice given un-deacidified extract A and C were 80% and 90%, respectively, at a volume of 0.2 mL/10 g BW. A few minutes after oral administration, both male and female mice showed similar symptoms of less movement, depression, unsteady gait, piloerection, and salivation. Half of the mice died within 30 min, and the other mice died within 24 h. The death rate decreased when a lower dose of extract A was given. In contrast, even with lower dose, the mortality of mice given extract C was still as high as 90 percent. This result also pointed to the reason as high lignan content.

Furthermore, comparing the toxicity data of extract A and B, the mortality of mice given B was not observed under all doses. Considering their low lignan content, the main difference lied in the organic acid content. After deacidification procedure, the organic acid content decreased more significantly in B than A, which can be regarded as a proof of the toxicity of organic acid under dosage used in this study.

Citric acid is also a common food additive and a generic component in fruits and vegetables, regarding their contents and daily consumption amount. However, the content in dried SCF reaches as high as 18% [5], which is quite rare, and also makes it hard to accept when orally administrated.

Based on the results of toxicity, the deacidification procedure improved the safety of ethanol extract by decreasing the content of organic acid and lignan compounds, and might facilitate the comprehensive utilization of SCF extract in food and beverage industries.

## 3. Materials and Methods

### 3.1. Sample Collection

*Schisandra chinensis* fruits (water content 12.6%) were purchased in Guoda TCM pharmacy (Shenyang, China) and were verified as the dried ripe fruits of *S. chinensis* (Turcz.) Baill by Associate Professor Jia Lingyun in Shenyang Pharmaceutical University. The specimen was stored in the herbarium of Shenyang Pharmaceutical University. The voucher specimen number is SYPU2016722-15. The dry fruits were ground into powder and passed through a sieve (50 meshes) before use.

### 3.2. Chemicals

Standard compounds (chlorogenic acid, schisandrin, Schisantherin A, deoxyschizandrin, and γ-schizandrin) were all purchased from the National Institute for the Control of Pharmaceutical (Beijing, China) and Biological Products of China. 2,2-Diphenyl-1-picrylhydrazyl (DPPH) was purchased from Sigma (Shanghai, China). HPLC-grade acetonitrile (ACN) was from J & K Scientific (Tianjin, China). Tris-HCl, citric acid, and *Folin-Ciocalteu’s* phenol reagent were purchased from Sango Biotech (Shanghai, China) Co. Ltd. Phosphoric acid, sodium carbonate, calcium carbonate, and 95% ethanol were of analytical grade and were ordered from Shandong Yuwang Chemical Company (Dezhou, China).

### 3.3. Sample Preparation

**Figure 4:**
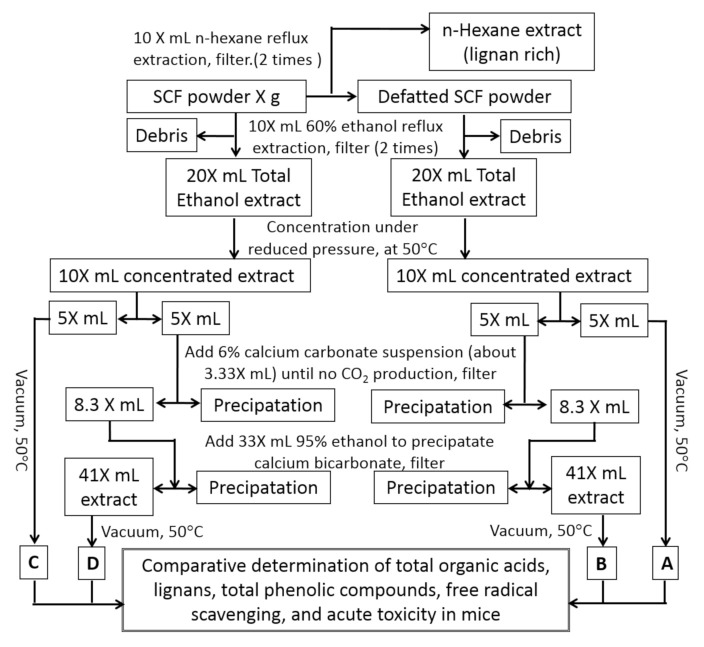
Scheme for the preparation of samples A, B, C, and D. Notes: Samples A and B are derived from defatted SCF powder (extracted with hexane; dashed lines); samples C and D are derived from SCF powder without hexane extraction. X represents the weight of SCF used in grams.

Deacidification of SCF ethanol extract was based on the published literature [20] with modification. As illustrated in Figure 4, to prepare the defatted *S. chinensis* samples A and B, 3.0 L of hexane was added to 300 g SC powder and mixed well. The mixture was subjected to reflux extraction for 2 h. The extract was filtered and the debris was extracted again with 3.0 L hexane. After filtration, the debris was dried in a hood to remove residual hexane and referred to as defatted SCF powder.

3.0 L 60% ethanol was added to the defatted SC powder obtained as described above. After thorough mixing, the solution was left at room temperature for 1 h, then subjected to reflux extraction for 2 h. The extract was filtered and the debris was extracted again with 3.0 L 60% ethanol. The two extracts were combined and mixed, giving a total of about 6.0 L of defatted extract. Half of the extract (3.0 L) was concentrated directly under vacuum at 50 °C to give sample A (defatted ethanol extract). The other half was concentrated to half volume and subjected to a deacidification process, as described in Figure 1, to give sample B (defatted, deacidified ethanol extract).

To prepared the non-defatted SC samples (C and D), 3.0 L 60% ethanol was added to 300 g SC powder and mixed well. The solution was left at room temperature for 1 h, and then subjected to reflux extraction for 2 h. The extract was filtered and the debris was extracted again with 3.0 L 60% ethanol. The two extracts were combined to give about 6.0 L of non-defatted extract. Half of the extract (3.0 L) was concentrated directly under vacuum at 50 °C to give sample C (untreated ethanol extract), while the other half was concentrated to half volume and subjected to a deacidification process, as described in Figure 1, to give sample D (deacidified ethanol extract).

The four samples (A, B, C and D) were dissolved in 60% ethanol to give a final concentration of 0.4–2%. The solutions were filtered through 0.45 μm filters and stored for subsequent HPLC and total phenolic assays.

### 3.4. Determination of Total Phenolic Compounds

*Folin-Ciocalteu’s* phenol reagent [30] was used to determine the total phenolic compound content, which was expressed as chlorogenic acid equivalence. Specifically, *Folin-Ciocalteu’s* phenol was diluted to 50% using distilled water. Sodium carbonate was dissolved in distilled water to a final concentration of 2%. 30 μL of each sample solution was added to 600 μL of sodium carbonate solution, mixed well, and incubated for 5 min. 30 μL of diluted *Folin-Ciocalteu’s* phenol was then added, mixed well, and incubated for 30 min. The absorbance at 750 nm was recorded. 30 μL of 60% ethanol was added and used as the blank. Chlorogenic acid was dissolved in 60% ethanol and diluted to different concentrations, and 30 μL of each diluted chlorogenic acid solution was used in the assay. The absorbance (X) and concentration of standard compound (Y) was correlated, and a standard equation was calculated as Y = 0.7018X − 0.0298 (R^2^ = 0.9954, linear range: 0.051–0.312 μg/μL).

### 3.5. DPPH Free Radical Scavenging Activity Assay 

Free radical scavenging activity was assayed based on the methods described in the publications. [23,31] A 0.2 M Tris-HCl buffer (pH = 7.5) was used to prepare an 80% ethanol solution. DPPH was dissolved in the prepared 80% ethanol solution to a final concentration of 0.05 mg/mL. In the free radical scavenging activity assay, 600 μL of DPPH solution was added to a 1.5 mL tube, followed by 30 μL of the sample solution. The solutions were mixed well and incubated in the dark at room temperature for 50 min. The absorbance at 517 nm was recorded as A_s_. 80% Tris-HCl ethanol solution mixed with 30 μL 60% ethanol was used as a blank, and A_0_ was recorded when 600 μL DPPH solution was mixed with 60% ethanol after incubation under the former conditions.

To compare the free radical scavenging activity of the four extract samples, the vitamin C (Vc) equivalence of each sample was calculated. Vc was dissolved in 60% ethanol solution and diluted to different concentrations, then 30 µL of each dilution was mixed with DPPH solution and A_Vc_ at 517 nm was recorded. The scavenging rates (A_0_ − A_Vc_) (X) and Vc concentrations (Y) were correlated and the standard equation was Y = 0.0014X − 0.0018 (R^2^ = 0.9913, linear range: 0.0053–0.106 μg/μL). The standard equation was used to calculate the Vc equivalence of different samples.

### 3.6. Lignan Determination

The levels of the four main lignans in the different samples were determined using a L-2000 HPLC machine (Hitachi, Tokyo, Japan), equipped with a L-2455 DAD detector, a L-2200 auto-sampler, a L-2130 pump, an AT-330 column heater, and a D2000 Chemstation. The methodology was adopted from a previously published paper [23]. A Plastisic C_18_ reverse phase column (4.6 × 250 mm, 5 μm) was used. Analytical conditions were as follows: Flow rate 1.0 mL/min, column temperature 30 °C, detection wavelength 250 nm, scanning range 200–400 nm. Experimental conditions were based upon a previous report [23]. Solvent A was a 0.5% phosphoric acid in ACN and solvent B was a 0.5% phosphoric acid aqueous solution. Analyte separation was obtained using the following gradient for solvent A: 51% raised to 61% in 35 min, held at 61% until 45 min, raised to 77% in 20 min, raised again to 100% in 10 min, then reduced to 51% in 5 min and maintained at 51% for 5 min. The sum of the four lignan contents was referred to as the total lignan content. In the HPLC analysis, samples C and D were diluted 5 times, and then the same volume of diluted C and D was injected and analyzed together with undiluted A and B sample solutions. Standard equations of four lignan are as follows: Schisandrin: Y = 2 × 10^−6^ X + 0.0233 (R^2^ = 0.9906, linear range: 0.006–0.923 μg); Schisantherin A: Y = 1 × 10^−6^ X + 0.1697 (R^2^ = 0.9912, linear range: 0.057–7.382 μg); deoxyschizandrin: Y = 9 × 10^−7^ X + 0.0243 (R^2^ = 0.9907, linear range: 0.006–0.945 μg); and γ-schizandrin: Y = 1 × 10^−6^ X + 0.0444 (R^2^ = 0.9914, linear range: 0.013–1.925 μg).

### 3.7. Determination of Organic Acid Content Using Potentiometric Titration

To determine the organic acid content in the ethanol extracts, citric acid was used as a reference compound [32]. Citric acid was dissolved in distilled water to a final concentration of 26.55 mg/mL and titrated against 0.1001 M NaOH using a pH-25CW Microprocessor pH/mV Meter with an E-201-C combination pH electrode (Shanghai Lida Instrument Company, Shanghai, China) while stirring with a magnetic stirrer (IKA) at 200 rpm. The E-V and (Δ*E*/Δ*V*) − *V* curve was drawn using Excel software. The end-point of the titration was determined from the highest Δ*E*/Δ*V* value on the (Δ*E*/Δ*V*) − *V* curve and the steepest slope on the E-V curve. The results showed that 1 mL 0.1001 M NaOH was able to titrate 6.411 mg citric acid (MW = 192.14). Each *S. chinenesis* ethanol extract (A–D) was dissolved in distilled water to a final concentration of about 1 g/50 mL and filtered. The filtrate was used for titration. The volume of NaOH (0.1001 M) at the end-point was recorded, and the citric acid equivalence of each sample was calculated.

### 3.8. Maximal Tolerance Dose and Maximal Feasible Dose

SPF grade Kunming mice, (about 4 weeks old, 18–22 g) were supplied by the Experimental Animal Center, Shenyang Pharmaceutical University. All experiments and procedures were carried out according to the Regulations of Experimental Animal Administration issued by the State Committee of Science and Technology of China (SYPU-IACUC-S2016-12.26-104).

The animals were housed by sex and maintained on a commercial pellet diet with distilled water given ad libitum. Mice were kept in plastic cages in a room at 20 ± 2 °C and 50–70% relative humidity with a 12-h light/dark cycle.

After 1-week acclimation, the maximal tolerance dose and maximal feasible dose methods were adopted to evaluate the acute oral toxicity of A, B, C, and D. All mice were randomly divided into four groups: A, B, C, and D (n = 10, 5 males and 5 females). All animals were fasted overnight before treatment. Samples A, B, C, and D were administrated once at a volume of 0.2 mL/10 g BW. If no mice died in 24 h, a further 10 mice (5 males and 5 females) were treated at the same concentration at a volume of 0.3 mL/10 g BW. After each administration, all the animals were continuously observed for mortality, behavioral changes, and physical signs of toxicity for up to 14 days afterwards. If no mice died at a volume of 0.3 mL/10 g body weight, the maximal feasible dose was obtained. If mice died at a volume of 0.3 mL/10 g body weight, the maximal tolerance dose was obtained. Lower doses (0.15 and 0.1 mL/10 g BW) were also designed to test the toxicity of four extract.

### 3.9. Statistical Analysis

All experiments were triple replicated. Values in content and FRSA determination were expressed as mean ± SD. The Student’s *t*-test was applied in the significance determination of indices between different groups.

## 4. Conclusions

In the present study, a chemical deacidification process was applied in SCF ethanol extract to decrease the content of organic acid. The effects of this process on the contents of four representative lignan, total phenolic compounds, and antioxidant activity were also determined. Furthermore, the acute toxicity of four kinds of extract were also compared in mice by oral administration. Advances on acute toxicity of different components in SCF, including specific lignans, essential oils, and citric acid were reviewed. In accordance with these published results, our data proved that the deacidification procedure improved the safety of SCF ethanol extract by decreasing the content of lignan and organic acid.

The deacidification process significantly decreased the contents of organic acids and total lignans. This will facilitate the development of health foods or beverages from *S. chinensis*. This process also provides the possibility to recover natural citric acid from the calcium citrate precipitate obtained. Furthermore, the extra extraction using hexane will enrich lignans, which can be used in medicine development. In general, these processes will facilitate the production of various kinds of *S. chinensis* extract, meeting the needs of the development of health foods, food additives, beverages, and medicines.

## Figures and Tables

**Table 1:** Organic acid content determination of sample A, B, C, and D. SCF: *Schisandra chinensis Fructus*.

	g SCF/g Extract	Organic Acid (%)	Deacidification Rate (%)
A	4.10 ± 0.12	33.72 ± 2.07	--
B	5.43 ± 0.20	6.23 ± 0.50	86.29 ± 0.01
C	2.36 ± 0.21	36.47 ± 0.65	--
D	2.50 ± 0.13	4.47 ± 0.29	88.00 ± 0.02

**Table 2:** Levels of total phenolic compounds (TP), free radical scavenging activity (FRSA), total lignans (TL) and organic acids (OA) in the samples A, B, C, and D and the corresponding levels in the crude material (SCF).

	g SCF/g Extract	Total Lignan (%)	Lignan Loss Rate (%)	Total Phenolic (%)	Total Phenolic Loss Rate (%)	FRSA (Vc %)	FRSA Loss Rate (%)
A	4.10 ± 0.12	0.61 ± 0.04	---	3.30 ± 0.14	---	0.59 ± 0.03	---
B	5.43 ± 0.20	0.24 ± 0.03 ^a^	70.60 ± 0.05	3.18 ± 0.09	27.21 ± 0.04	0.69 ± 0.02	10.93 ± 0.06
C	2.36 ± 0.21	2.70 ± 0.13	---	5.07 ± 0.11	---	0.82 ± 0.02	---
D	2.50 ± 0.13	1.75 ± 0.22 ^a^	38.32 ± 0.13	2.37 ± 0.12 ^a^	55.92 ± 0.05	0.45 ± 0.05 ^a^	48.18 ± 0.11

Note: ^a^ represents the significance of *p* < 0.05 when indices in B were compared with that in A, or indices in D were compared with that in C.

**Table 3:** The published LD_50_, the content (%) in *S. chinensis*, and the calculated LD_50_ of the dried fruit (SCF) for various compounds and the ethanol extract. O. A., orally administered in mice.

Compound Name	LD_50_ g/kg BW O.A.	Content (%)	Calculated SCF LD_50_ (g/kg BW)
Ethanolic extract	7.15–9.74 [24]	48.78 [24]	14.67–19.96
Schisandrin	1.0 [25]	0.49–0.85 [26]	117–204
γ-schizandrin	0.25 [25]	0.1–0.36 [26]	69–250
Essential oils	8.75 ± 2.41 [27]	1.6 [28]	546 ± 150.6
Citric acid	5.040 [29]	18 [5]	28

**Table 4:** Acute toxicity test results of samples A, B, C, and D in mice by oral administration.

	0.3 mL/10 g BW		0.2 Ml/10 g BW		0.15 mL/10 g BW		0.10 mL/10 g BW	
	F	M	F	M	F	M	F	M
A	5/5 ^#^	5/5	4./5	4/5	3/5	4/5	2/5	1/5
B	0/5 *	0/5 *	0/5 *	0/5 *	-	-	-	-
C	5/5	5/5	5/5	4/5	4/5	4/5	4/5	5/5
D	4/5 ^##^	4/5 ^##^	0/5 **	0/5 **	-	-	-	-

^#^: The denominator shows the total number of mice used, and the numerator shows the number of dead mice. *: The significance of *p* < 0.001 between sample B and sample A. **: The significance of *p* < 0.001 between sample D and sample C. ^##^: The significance of *p* < 0.001 between sample D and sample B.
